# Symphony in the crowd: Key genetic alterations in prostate cancer

**DOI:** 10.1002/cai2.52

**Published:** 2023-02-09

**Authors:** Neshat Masud

**Affiliations:** ^1^ Department of Pharmacology University of Louisiana at Monroe Monroe LA USA

**Keywords:** prostate cancer, alterations, genetic, cancer, symphony

## Abstract

Androgen receptor (AR) signaling have been frequently targeted for treating prostate cancer (PCa). Even though primarily patients receive a good therapeutic outcome by targeting AR signaling axis, eventually it emerges resistance by altering the genetic makeup of prostate cells. However, to develop an effective therapeutic regime, it is essential to recognize key genetic alterations in PCa. The most common genetic alterations that give rise to distinct androgen different differentiation states are gene fusion of TMPRSS2 with ETS family genes, deletion, or mutation of tumor suppressor *PTEN* and *TP53* gene, amplification or splicing of AR, altered DNA repair genes. In this review, we describe key genes and genetic changes that have been recognized to contribute to altered prostate environment.

## Graphical abstract

Genetic alterations in prostate cancer (PCa). PCa progresses from a recurrent inflammation of the prostatic epithelia to an aggressive form. The key genetic alterations during this conversion include: altered androgen receptor (AR), gene fusion, alteration of tumor suppressors, defective DNA repair mechanism.

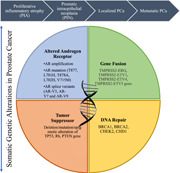

**Abbreviations**
ADT: androgen deprivation therapy
AR: androgen receptor
ERG: ETS‐related gene
ETS: E26 transformation specific
PCa: prostate cancer
PCA3: prostate cancer antigen 3
PIA: proliferative inflammatory atrophy
PIN: prostatic intraepithelial neoplasia
PSA: prostate‐specific antigen
PTEN: phosphatase and tensin homolog
TMPRSS2: transmembrane protease serine 2

## 1 INTRODUCTION

Prostate cancer (PCa) is one of the leading causes of cancer‐related death in men. In 2022, an estimate of 268,490 new cases were diagnosed and 34,500 deaths occured in the United States by PCa [1]. Despite this considerable number of cases, the pathogenesis and etiology remain a mystery for PCa treatment.

Race and family history have long been identified as a risk factor for PCa [2]. African American men possess the highest risk than those of Caucasian men, whereas Asian men possess the lowest risk of all. African American men also develops an early onset of PCa and a high mortality rate than men of any other origin. Moreover, men with a family history of PCa has twice the risk of developing PCa compared with those who lack a family history [3]. Complementing this finding, genome‐wide association studies have identified distinct loci on chromosomes, for example, 8q24, 8p21, 11q13, 17q21, and 17p12 responsible for susceptibility in PCa [4, 5, 6, 7]. Additionally, several literatures have published exciting studies regarding the association of single‐nucleotide polymorphisms with the aggressiveness in PCa development [5]. These evidence suggest a strong genetic basis for PCa.

PCa arises in the prostate gland. Prostate gland is a tubuloalveolar organ and can be divided into central, transitional, and peripheral zones. The peripheral zone contributes to >70% of adult prostate tissue and composed of multiple secretory acini and duct [8, 9]. The duct and acini consist of branching epithelial compartment. This epithelium compartment comprises three primary cell types: luminal, basal, and neuroendocrine cells. These cells are differentiated by their genetic makeup and cell‐specific markers (Table 1). Although there is controversy regarding the cell of origin of PCa, it is strongly believed that an accumulation of somatic alterations in these distinct differentiated cells is responsible for PCa development [10].

**Table 1:** Prostate‐specific markers and location in prostate cells.

Prostate‐specific marker	AR regulated	Cellular location	References
PSA	**√**	Cytoplasmic	[36]
AR	**√**	Nuclear/cytoplasmic	[37]
PSMA	**√**	Membrane	[37]
PSCA	**√**	Membrane	[37]
AMACR	No	Granular cytoplasmic	[37]
PSAP	**√**	Granular cytoplasmic/membranous	[37]
TMPRSS2‐ERG	**√**	Nuclear	[21]
PCA3	**√**	N/A (RNA)	[38]
Homeobox gene NKX3.1	**√**	Nuclear	[37]
HOXB13	No	Nuclear	[37]
KLK4	**√**	Cytoplasmic	[39]
Prostate secretory protein (PSP94)	**√**	Nuclear/cytoplasmic	[37]
KLK2	**√**	Cytoplasmic	[39]

Abbreviations: AMACR, ɑ‐methylacyl‐CoA racemase; AR, androgen receptor; ERG, ETS‐related gene; HOX‐B13, homeobox protein Hox‐B13; KLK2, human glandular kallikrein; KLK4, human kallikrein‐related peptidase 4; PCA3, prostate cancer antigen 3; PSA, prostate‐specific antigen; PSAP, prostate‐specific acid phosphatase; PSCA, prostate stem cell antigen; PSMA, prostate‐specific membrane antigen.

The secretory luminal cells express a high level of AR and secrete prostate differentiation markers (e.g., prostate‐specific antigen [PSA]). These cells respond to androgen and maintain AR signaling. Hence, androgen‐mediated translocation and activation of AR have been considered as an integral part for the growth, development, and maturation of prostate. This mechanism is evident during embryonic stage, whereas fetal testicular secretion of androgen hormone causes prostatic development. In later years, adult prostate requires androgen and AR activation activity to maintain prostate function and reproductive activity [11]. Androgen deprivation inhibits the morphogenesis of prostate gland [12]. Therefore, therapeutic strategy has been developed to deprive prostate cells of androgen known as androgen deprivation therapy (ADT). Many patients respond well during the initial course of ADT treatment. However, a majority of PCa progresses to a more aggressive phonotype following 2 years of ADT. The conversion from a slow growing indolent localized tumor to an aggressive phenotype involves a wide array of changes that result in defective cell regulatory mechanisms ultimately leading to genetic structural rearrangements. To understand the divergent nature of PCa and develop advanced treatment options, it is necessary to recognize key genetic changes that lead to prostate tumor development.

## 2 SIGNIFICANCE OF IDENTIFYING KEY GENETIC ALTERATIONS

**Figure 1:**
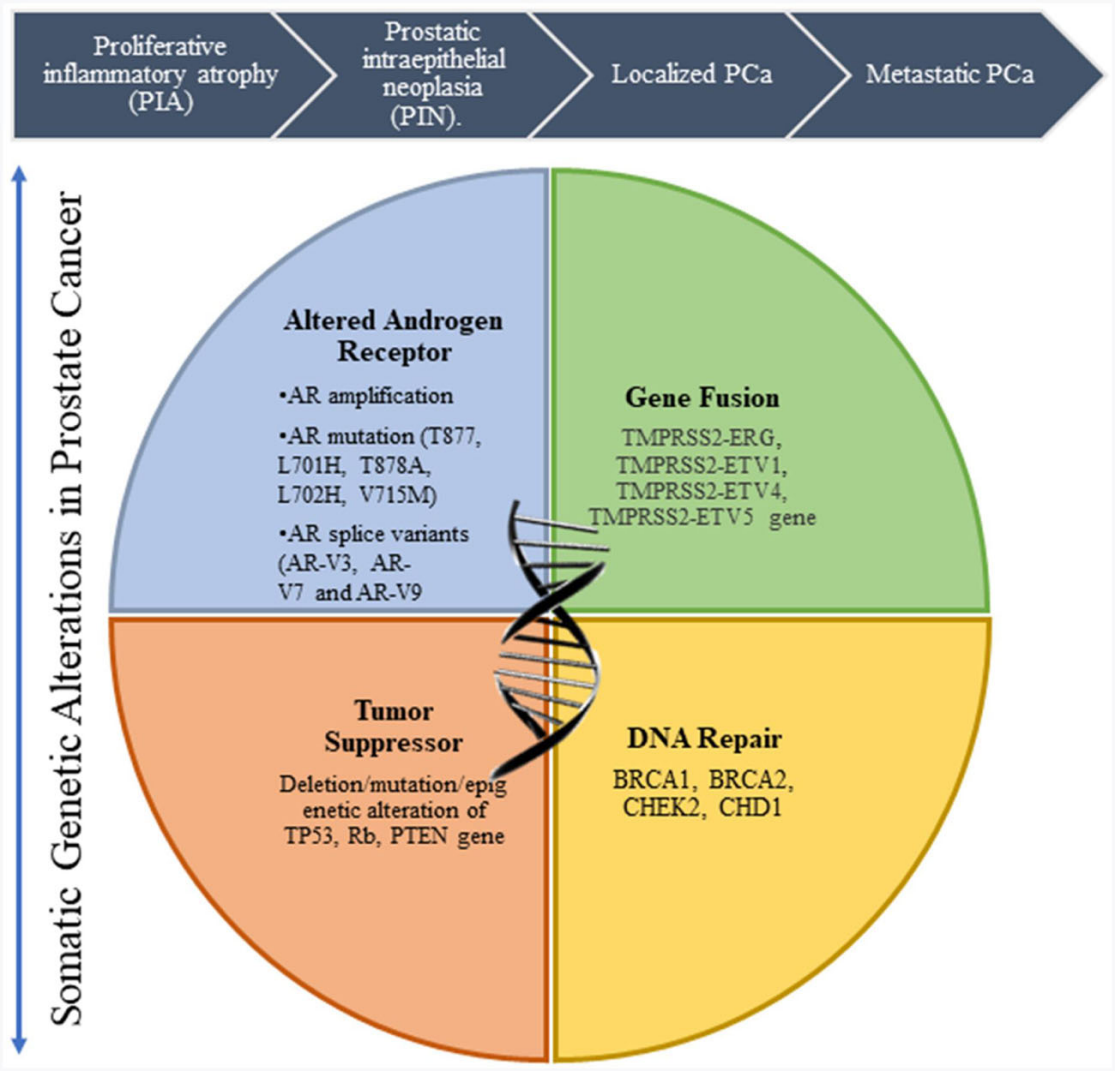
Genetic alterations in prostate cancer (PCa). PCa progresses from a recurrent inflammation of the prostatic epithelia to an aggressive form. The key genetic alterations during this conversion include: altered androgen receptor (AR), gene fusion, alteration of tumor suppressors, and defective DNA repair mechanism.

Malignant transformation of the prostatic epithelia involves a complex series of biological and molecular events that are initiated by genetic changes. Prostate tumor starts with a recurrent inflammation known as proliferative inflammatory atrophy (PIA) (Figure 1). PIA is also considered as a morphological precursor of prostatic intraepithelial neoplasia (PIN). PIN is followed by localized PCa and then culminating in metastatic PCa [13]. The genetic landscape of metastatic PCa harbors a high degree of genetic alterations and heterogeneity compared to localized PCa [14]. This heterogeneity of PCa can be useful for identifying distinct genes in different stages of PCa, which could lead to effective genomic targeting during this malignancy. Even though PCa is considered as an age‐related disease, studies of prostate specimen from healthy men from age 20 to 40 years demonstrated the presence of histologic foci [15]. It suggests that the onset of PCa starts at an early age. However, the deficiency of proper marker and treatment regime makes it difficult to prevent PCa. However, identification of key genetic alterations can signify different stages of PCa.

Currently, the established biomarker for detection of PCa is PSA, which is misleading due to low specificity. PSA level can exhibit a very low level (<2.5 ng/mL) despite having very aggressive PCa. Alternatively, a high level of PSA (>10 ng/mL) can be ambiguous despite benign stage. Moreover, the traditional PCa detection strategy—prostate biopsy—can be very uncomfortable and painful to patients. In addition, sample collection from central or transitional zone or any other tumor null environment can exhibit false negative disease. Due to these limitations, it is extremely urgent to detect key genetic modifications for diagnosis and prognosis of PCa.

The good news is that the breakthrough of discoveries such as genome‐wide analysis, microarrays, and high‐throughput sequencing have enabled us to identify key genes that play an important role for the divergent nature of PCa. In this review, we describe key genes and genetic events for the pathogenesis and prognosis of PCa.

### 2.1 *TMPRSS2:ETS* gene fusion

Even though recurrent gene fusion occurs at a low frequency in different solid tumors, prostate tumors harbor a frequent phenomenon of gene fusion. Recent studies reported that AR is responsible for this gene fusion mechanism in PCa [16]. AR activates certain oncogenes by recruiting enzymes, for example, topoisomerase II β, cytidine deaminase, ORF2 endonuclease, or by reprogramming the binding elements of that target gene [16, 17, 18]. Among these mechanisms, transmembrane protease serine 2 (TMPRSS2) gets activated by reprogramming of the intronic regions and is located to a proximity of erythroblastosis virus E26 transformation specific (ETS)‐related gene (ERG). *TMPRSS2* is an AR‐regulated gene and erythroblast transformation‐specific (ETS) is a transcription factor that recognizes GGAA/T sequence as binding sites in the target genes. Through this binding, ETS acts as an activator or repressor of transcription and plays a major role as a driving oncogene in androgen‐mediated *TMPRSS2‐ETS* gene fusion [19]. TMPRSS2 and ETS, both are located on chromosome 21 within a range of ~3 Mb. *TMPRSS2‐ETS* gene fusion have been reported in more than 50% of prostate tumor cases [20]. The gene fusion event usually involve the 5′‐untranslated gene promoter region of TMPRSS2 with the 3′‐exon of ETS transcription factors, ERG or ETV1 [21, 22]. Several investigations showed that androgen stimulates *TMPRSS2‐ETS* gene fusion. Tomlins et al. [21] identified that androgen stimulates ∼2000‐fold ERG expression in *TMPRSS2‐ETS* gene fusion‐positive VCaP PCa cell line compared with *TMPRSS2‐ETS* gene fusion‐negative LNCaP PCa cells. More interestingly, the study identified that when a combination of AR antagonist is used with synthetic androgen, VCaP cell line exhibits an increase in ERG expression [21]. These results suggested that *TMPRSS2‐ETS* gene fusion is more prevalent in an aggressive form of PCa than normal, benign, and androgen‐responsive phenotype. This signifies the biggest advantage of using *TMPRSS2‐ETS* gene fusion for the detection of PCa as normal prostate lacks this fusion. *TMPRSS2‐ETS* gene fusion is also related to poor clinical outcome in patients. Loss of certain genomic regions of *TMPRSS2‐ETS* gene known as “class Edel,” exhibited less survival in patients [23]. Even though *TMPRSS2‐ETS* gene fusion is not a tumor progression marker but due to its relevance with aggressiveness, *TMPRSS2‐ETS* gene fusion remains a curious novel target for therapeutic development in PCa.

### 2.2 Loss of phosphatase and tensin homolog (*PTEN*) gene

*PTEN* gene loss frequency correlates positively with an increase in Gleason score [24]. In Gleason score 7, loss of PTEN is twice as frequent than that of Gleason score 6. Hence, PTEN is considered as an early marker for the detection of PCa. PTEN acts as a lipid phosphatase inhibiting the activity of phosphatidylinositol 3‐kinase (PI3K) and reducing the level of serine/threonine kinase, Akt, a mechanistic target of rapamycin. Thus, loss of PTEN results in an increase in Akt‐pathway, which promotes the survival of cancer cells. In most solid tumors, PTEN is inactivated by heterogenous deletion, whereas homozygous deletion of the *PTEN* gene is a unique characteristic in PCa [25]. Evidence showed that in mice, monoallelic deletion of *PTEN+/−* gene resulted in the formation of PIN. In contrast, biallelic deletion of *PTEN−/−* gene resulted in invasive and metastatic prostatic adenocarcinoma [24, 26]. Genetically engineered mouse model studies have uncovered the association of PTEN loss with other key genes that are frequently altered in prostate tumor microenvironment. Studies reported that PTEN loss occurs after TMPRSS2‐ETS rearrangement and it signifies a correlation between ERG expression and loss of *PTEN* gene [27]. Interestingly, PTEN loss is enriched two‐ to fivefolds higher in localized prostate tumors with ERG rearrangement. ERG expression restores AR program and possibly this could be the mechanism for the re‐expression of AR in castration‐resistant PCa development. However, loss of PTEN could also account for loss of Tp53. Concomitant loss of tumor suppressor PTEN and Tp53 results in metastatic progression of PCa. In addition, PTEN can also be inactivated by epigenetic alteration presumably by promoter methylation. In a clinical study, PTEN expression was restored by the use of epigenetic demethylating agent, which was suggesting that PTEN is inactivated by methylation in addition to deletion or mutation [28]. PTEN promoter methylation significantly correlates with proliferation of cancerous cells and metastatic progression of the disease [28]. Preclinical studies demonstrated that inhibition of PI3K‐Akt pathway restores *PTEN* gene and AR signaling pathway. Thus, simultaneous targeting of AR pathway and PI3K‐Akt pathway could be beneficial for treating PCa and restoring *PTEN* gene.

### 2.3 AR gene alteration

AR signaling is the mainstay therapeutic element for treating PCa. AR mediates physiologic effects by binding to androgens. Upon the binding of androgens to AR, AR‐dimer translocate to nucleus, binds to androgen response elements, and initiate transcription of target genes. This transcription promotes the growth, development, and maturation of normal prostate. Considering the functional significance of AR, research and therapeutics have been developed targeting AR. However, cumulative evidence suggests that a great majority of AR inhibition still maintains AR signaling. Even though this mechanism is not clear, AR gets reactivated in response to any steroidogenic agents or alteration of AR gene. Among these AR gene alterations, AR mutation, AR overexpression, and AR splicing have gained much clinical significance. Generally, AR is expressed at a low level in normal prostate but when patients are treated with ADT, AR expression gets increased (20%–30%) higher than normal prostate. Enhanced AR expression sensitizes prostate to low level of androgens, thus stimulating continuous prostate growth [29]. Point mutation of AR is PCa was observed in several patients when they were treated with anti‐androgens. AR point mutations such as T877A, L701H, T878A, L702H, and V715M were further confirmed in several studies [27, 28]. The latest advancement for the resistance mechanism in PCa is the splicing of AR. These splice variants are generated by the rearrangement of the exon or complete removal of the ligand‐binding domain or DNA‐binding domain of AR. Studies reported that an increased level of the messenger RNA of splice variants such as AR‐V3 and AR‐V9 causes metastatic progression of PCa [30]. Enhanced expression of AR‐V7 increases the possibility of castration‐resistant PCa [31]. Even though AR is not prostate specific, alteration of AR certainly plays a major role in the cancerous progression of PCa.

### 2.4 Altered DNA repair genes

Genome‐wide studies revealed that 15%–35% of castration‐resistant PCa exhibits defects in DNA repair genes (The Cancer Genome Atlas Research Network, 2015). Mutations of BRCA1 and BRCA2 are associated with aggressive phenotype of PCa [32, 33]. *CHD1* gene is associated with chromatin remodeling and DNA repair by recruiting proteins in response to DNA double‐strand break. Five percent to 10% PCa cases display loss of *CHD1* gene. Loss of CHD1 correlates positively with the loss of *PTEN* gene and fusion of *TMPRSS2‐ETS* gene [34, 35]. A more thorough investigation of DNA repair genes with AR alteration, loss of PTEN, and *TMPRSS2‐ETS* gene fusion could help in the therapeutic development in PCa.

## 3 CONCLUSION

The conversion of PIN to prostate tumor involves an array of genetic and epigenetic changes. Based on the gene and genetic environment, different biological mechanisms contribute to this transformation such as activation of cross‐signaling pathway, alteration in transcription factors, genomic rearrangements, and/or epigenetic reprogramming. These are not the only mechanisms, but several studies reported, these mechanisms have an intricate relationship with PCa development.

An important and complicated issue in PCa is the detection and treatment strategy. PCa starts with an androgen‐dependent state. During androgen‐dependent state, PCa patients receive androgen ablation therapy either through surgical castration or pharmacological mechanism. Possibly after quite a few months to 2 years, PCa emerges an androgen‐independent form. Although there are emerging research and upgraded technologies identifying novel genes to describe this sudden phenotypic change, conversion of PCa still remains a mystery for modern science. However, pinpointing key genes and genomic events can provide a significant insight on PCa development and promises advanced therapeutic regime in PCa.

## AUTHOR CONTRIBUTIONS

Neshat Masud has contributed to the design, editing and revision of the manuscript.

## CONFLICT OF INTEREST STATEMENT

The author declares no conflict of interest.

## ETHICS STATEMENT

This article does not violate any ethical standards.

## INFORMED CONSENT

Not applicable.

## Data Availability

Data sharing not applicable – no new data generated.
